# Genetic features of *Orthonairovirus haemorrhagiae* variants detected in ixodid ticks in Armenia

**DOI:** 10.1371/journal.pntd.0013752

**Published:** 2025-11-21

**Authors:** Anna Volynkina, Yana Lisitskaya, Anna Zhirova, Olga Gnusareva, Ekaterina Vasilenko, Lyudmila Shaposhnikova, Sergey Pisarenko, Arsen Manucharyan, Gayane Melik-Andreasyan, Vladimir Dedkov, Anna Dolgova, Alexander Kulichenko

**Affiliations:** 1 Stavropol Research Antiplague Institute, Federal Service for the Oversight of Consumer Protection and Welfare, Stavropol, Russia; 2 National Center for Disease Control and Prevention, Armenian Ministry of Health, Yerevan, Armenia; 3 Saint Petersburg Pasteur Institute, Federal Service for the Oversight of Consumer Protection and Welfare, Saint Petersburg, Russia; 4 Martsinovsky Institute of Medical Parasitology, Tropical and Vector Borne Diseases, Sechenov First Moscow State Medical University, Moscow, Russia; Public Health Agency of Canada, CANADA

## Abstract

**Introduction:**

Crimean-Congo hemorrhagic fever (CCHF) is a viral transmissible febrile disease, featuring hemorrhagic manifestations and a high mortality rate, caused by *Orthonairovirus haemorrhagiae* (CCHFV). CCHF is endemic in the Republic of Armenia (RA) and neighboring countries. Currently, there is no genetic information on CCHFV circulating in the RA. This work presents the results of genetic identification of CCHF viral variants detected in ixodid ticks collected in 2022–2023.

**Methodology:**

Ixodid ticks were collected from cattle, horses, and vegetation in seven Armenian regions (Vayots Dzor, Gegharkunik, Kotayk, Lori, Syunik, Tavush, Shirak). Tick pools were tested for the presence of CCHFV RNA by RT-PCR. Genetic identification of CCHFV isolates was performed based on partial and complete genomic segment sequencing (S, M, L).

**Results:**

Of 860 total tick pools, 77 were positive for CCHFV RNA. Such RNA was detected in *Hyalomma marginatum* (n = 71), *Haemaphysalis punctate (n = 2), Ixodes ricinus (n = 2), Rhipicephalus bursa (n = 2)*. CCHFV circulation was confirmed in the Syunik and Tavush regions. Phylogenetic analysis based on partial and full-length genomic segments showed that CCHFV strains of the Europe 1 and Europe 3 lineages circulated in the RA. Within the Europe 1 lineage, Armenian viral isolates belonged to different genetic subgroups: Vb, strains of which are widespread in the south of the European region of Russia; and new subgroups (Armenia-1, Armenia-2). Genomic segment reassortment events were revealed in the analyzed CCHFV sequences.

**Conclusion:**

This study provides new data on CCHFV genetic diversity in the RA. Further genetic studies of CCHFV circulating in countries of the Transcaucasian region are necessary to reconstruct the temporal and spatial viral distribution.

## Author summary

Crimean-Congo hemorrhagic fever (CCHF) is one of the most severe viral zoonotic diseases in humans. *Orthonairovirus haemorrhagiae* (Crimean-Congo hemorrhagic fever virus, CCHFV) is one of the most genetically heterogeneous arboviruses, and eight main genetic lineages have been described. Different viral genetic lineages and subtypes circulate in distinct regions. CCHF is endemic in the Republic of Armenia (RA) and bordering countries, wherein CCHF cases are registered annually. There is currently no data on CCHFV genetic diversity in the RA. As a result of this work, new data on viral genetic variants in the RA has been provided. The data may be used in epidemiological analysis of CCHF cases in the RA and other countries to differentiate local and imported cases.

## Introduction

Crimean-Congo hemorrhagic fever virus (CCHFV, *Orthonairovirus haemorrhagiae*) belongs to the genus *Orthonairovirus*, family *Nairoviridae*. It is the causative agent of Crimean-Congo hemorrhagic fever, which is a viral transmissible febrile disease, with hemorrhagic manifestations and a high mortality rate, reaching 30% in some regions [1,2]. The CCHFV genome consists of three segments of single-stranded, negative-sense RNA: S (1672 nt), M (5364 nt), and L (12150 nt). The organization is as follows: the S segment encodes nucleoprotein (NP); the M segment encodes glycoprotein precursor (GPC); and the L segment encodes RNA-dependent RNA polymerase (RdRp) [3]. CCHF viral strains are divided into eight main genetic lineages based on genomic segment nucleotide sequence: Africa (1,2,3); Asia (1,2); and Europe (1,2,3). Lineage names correlate with the geographic distribution [4,5]. Viral isolates form genetic subgroups within the main lineages based on circulation in different countries and local populations [6].

Ixodid ticks of the genus *Hyalomma* are the main vector and reservoir of CCHFV, although other tick species (genera *Rhipichephallus, Dermacentor*) are also involved in viral circulation. Natural CCHF foci are located in Africa, Southern Europe, Eastern Europe, and Asia, where ticks of the genus *Hyalomma* are common [7].

The Republic of Armenia (RA) is part of the South Caucasus region (Transcaucasia), located at the junction of Eastern Europe and Southwest Asia. The RA is bordered by Georgia (to the north), Azerbaijan (to the northeast, east and southeast), Iran (to the south), and Turkey (to the west). In neighboring countries, active CCHFV circulation leads to CCHF incidence registered annually [8]. According to ProMED-mail, from 2015-2024, about 1,000 confirmed CCHF cases were detected in Turkey per year, and from 14 to 150 cases per year were registered in Iran. From 2009-2022, 164 human CCHF cases were identified in Georgia. Circulation of certain viral genotypes (Asia 1, Asia 2, Europe 1, Europe 3) has been identified in Iran [9,10], and two genotypes (Europe 1, Europe 2) have been detected in Turkey [11].

In the Republic of Armenia, CCHFV has been detected in ticks from 1968-1999 [12,13]. The only human case of CCHF in Armenia was reported in 1974 [14]. Several factors contribute to maintaining the activity of CCHF natural foci in the RA. These are: climatic and environmental conditions; the presence of tick species suitable as viral vectors; and the presence of vertebrate hosts suitable as reservoir [15]. Currently, there is insufficient information about the rate of CCHFV infection among ixodid ticks in the region. There is no data available on genetic characteristic of CCHFV circulating in the RA. The aim of this work was specific viral RNA detection in ixodid ticks collected in the RA (2022–2023), including genetic characterization of viral variants.

## Materials and methods

### Ethics statement

The author confirms that all applicable international, national and/or institutional guidelines for the care and use of animals were followed. The author declares that only tick samples were collected from live animals and no other manipulations such as blood collections was undertaken on these animals for this study. The tick collection protocol from animals was approved by Local Ethics Committee of Stavropol Research Antiplague Institute.

### Study area

The study was conducted in 2022–2023 (from June to August) in seven Armenian regions (Vayots Dzor, Gegharkunik, Kotayk, Lori, Syunik, Tavush, Shirak). Tick collection and counting for laboratory testing were performed.

### Tick pools

A total of 3,938 tick specimens, belonging to 16 species, were collected from cattle, horses, and vegetation using the flagging method. Species were *Dermacentor marginatus, D. niveus, D. reticulatus, Haemaphysalis caucasica, H. concinna, H. erinacei taurica, H. parva, H. punctate, H. sulcate, Hyalomma asiaticum, H. marginatum, Ixodes laguri, I. ricinus, Rhipicephalus annulatus, R. bursa*, and *R. sanguineus*. Ticks were identified based on morphological characteristics and sorted by species, sex, development stage, collection date, and sampling site. Pools were formed containing up to 50 unfed adult ticks, or up to 5 half-fed adult ticks. Fully fed ticks were tested individually. Ticks in pools were washed in 70% ethanol, rinsed in sterile 0.15 M NaCl solution, and homogenized in 1 ml of 0.15 M NaCl solution. Tick suspensions were stored at -70°C.

### Detection of viral RNA

The qPCR screening for presence CCHFV RNA was performed using the RT-PCR CCHFV-FL Kit (AmpliSens, Russia). Viral RNA was extracted from tick suspensions using the RIBO-prep extraction kit (AmpliSens, Russia). Internal control RNA template (provided with the kit) was added to each sample. Complementary DNA was obtained using the REVERTA-L kit (AmpliSens, Russia). The viral load of positive samples was tested by qPCR using the PCR CCHFV-FL Kit (AmpliSens, Russia). Recombinant plasmid (containing target CCHFV genomic sequences) was serially diluted and used to create a standard curve for calibration (10^5^ to 10^1^ copies per reaction). The estimated viral load in 1 ml of tick suspension was 50-fold higher than in the reaction.

### Partial and full-length CCHFV genome sequencing

Genetic identification of CCHF viral isolates was performed based on partial and complete genomic segment (S, M, L) sequencing. Fragments of S segment (position 115–652), M segment (position 4620–5075), and L segment (position 105–541) were amplified using corresponding primer pairs: S-100f and S-680r; 24 and 25; and L-100f and L-540r [5]. Complete S, M, and L segments of the CCHFV genome were amplified as 20 overlapping fragments (amplicon lengths 650–1465 bp) using a primer set [16]. Amplified PCR products were purified using the CleanMag DNA PCR Kit (Evrogen, Russia). Nucleotide sequence determination was performed by Sanger sequencing on the SeqStudio genetic analyzer (ThermoFisher, USA) using the Big Dye Terminator Kit (v.3.1).

### Phylogenetic and geographic analysis

Contigs were assembled in Vector NTI 8.0 software. CCHFV isolate sequences with no ‘mixed peaks’ were used for analysis. Partial and complete nucleotide sequences of CCHFV strains obtained in this study were compared with reference segment (S, M, L) sequences of CCHFV isolates belonging to different genetic lineages available in GenBank (S1 Table). Multiple alignment of nucleotide and amino acid sequences was performed using the DECIPHER package of the R language (version 4.2.2). Phylogenetic analysis was conducted via the neighbor-joining method according to the Kimura-2 algorithm using Mega 11.0.13 software. The statistical significance of the phylogenetic tree topology was assessed by bootstrap test with 1000 replications. The coordinates of the ixodid tick collection site were linked to the electronic map of the Republic of Armenia using the QGIS 3/34/3 program. Basemap shapefiles were sourced from GADM (https://geodata.ucdavis.edu/gadm/gadm4.1/shp/).

## Results

### Species composition and ixodid tick abundance

Ticks (3,938 specimens) belonging to 16 species were collected from cattle, horses, and vegetation in seven regions: Vayots Dzor, Gegharkunik, Kotayk, Lori, Syunik, Tavush, and Shirak (Table 1). *H. marginatum* accounted for 49.1% of all ticks collected in 2022–2023. This species was found in six Armenian regions and predominated in the Gerakunik, Syunik, and Shirak districts. In the Syunik and Tavush regions, *H. asiaticum* ticks were also detected (0.4% and 1.1% of all ticks in region). *R. bursa* ticks were found in five regions, with regional prevalence from 0.2 to 77.4%.

**Table 1:** Viral RNA (CCHFV) detection in ixodid ticks collected in Armenia (2022-2023).

No	Tick species	Tick specimens		Tick pools	CCHFV^+^ pools	
		number	%	number	number	%
1	*Dermacentor marginatus*	277	7.03	51	0	0
2	*Dermacentor niveus*	156	3.96	26	0	0
3	*Dermacentor reticulatus*	143	3.63	30	0	0
4	*Haemaphysalis caucasica*	1	0.03	1	0	0
5	*Haemaphysalis concinna*	6	0.15	2	0	0
6	*Haemaphysalis erinacei taurica*	3	0.08	1	0	0
7	*Haemaphysalis parva*	6	0.15	1	0	0
8	*Haemaphysalis punctata*	69	1.75	37	2	5.4
9	*Haemaphysalis sulcata*	1	0.03	1	0	0
10	*Hyalomma asiaticum*	14	0.36	11	0	0
11	*Hyalomma marginatum*	1934	49.11	449	71	15.8
12	*Ixodes laguri*	20	0.51	5	0	0
13	*Ixodes ricinus*	122	3.10	40	2	5.0
14	*Rhipicephalus annulatus*	351	8.91	53	0	0
15	*Rhipicephalus bursa*	752	19.10	134	2	1.5
16	*Rhipicephalus sanguineus*	83	2.11	18	0	0
	TOTAL	3938	100.00	860	77	8.9

A total of 203 farm animals (4 horses, 199 cattle) were examined for the presence of ixodid ticks. The predominant tick species found on farm animals were *H. marginatum* (78.7%) and *R. bursa* (13.2%). Overall, 94% of inspected animals were infested with ixodid ticks, and 81.1% of cattle were infested by *H. marginatum,* which is the main vector of CCHFV. The abundance index of adult *H. marginatum* ticks was 10.5 in 2023 and 3.2 in 2022. The proportion of female *H. marginatum* in cattle was 16–31%; the proportion of males was 69–84%.

In some farms in the Syunik and Kotayk districts, a high number of *R. bursa* ticks on cattle was observed (infestation rate 78%, abundance index 4.4). This, along with *H. marginatum* ticks simultaneously feeding on the same host animal, increases the probability of *R. bursa* ticks being involved in CCHFV transmission and circulation.

### Viral RNA detection in ixodid ticks

Ixodid tick pools (n = 860) collected from Armenian regions were tested for the presence of CCHFV RNA by qPCR. Seventy-seven tick pools (8.9%) were positive. The indicated CCHF-positive samples (Table 1) were found within the pools of *H. marginatum* (n = 71), *H. punctata* (n = 2), *I. ricinus* (n = 2), and *R. bursa* (n = 2). All positive ixodid tick pools were collected from cattle in June-July, 2022–2023.

Ticks positive for CCHFV RNA were found in two regions. Syunik had 76 pools in 2022–2023 (*H. marginatum, H. punctata, I. ricinus, R. bursa*). Tavush had 1 pool in 2023 (*H. marginatum*). In the Syunik region, the prevalence of CCHFV infected *H. marginatum* samples was 23.6% in 2022 and 28.1% in 2023. In the Tavush region, it was 33.3% in 2023.

**Fig 1:**
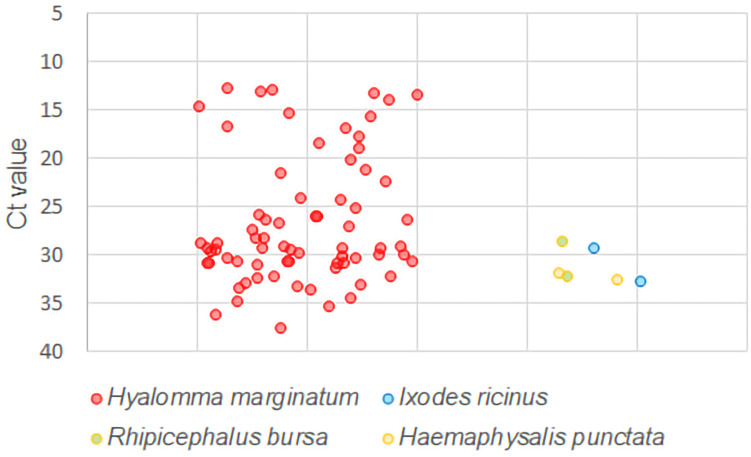
Comparison of Ct values for *H. marginatum* and other tick species.

As shown in Fig 1, higher CCHFV viral loads were found in *H. marginatum* tick pools (Ct 12.46 – 37.58, viral load 2 × 10^8^ – 5 × 10^3^ copies/ml of tick suspension) than in pools belonging to *H. punctata*, *I. ricinus*, and *R. bursa* (Ct 29.28 – 32.45, viral load 5 × 10^4^ – 1 × 10^4^ copies/ml of tick suspension). An independent t-test was used for comparison of Ct value between *H. marginatum* and other tick species groups. The group differences were significant (*p* = 0.0002 with *p* < 0.05 considered significant). *H. marginatum* ticks with high viral RNA loads (Ct 12.46 – 16.81, viral load 2 × 10^8^ – 3 × 10^7^ copies/ml of tick suspension) were also detected on the farm animals from which CCHFV^+^ *H. punctata*, *I. ricinus*, and *R. bursa* ticks were collected.

### CCHFV genetic identification based on partial genomic segments

Partial S, M, and L segment sequences of CCHFV isolates from nine *H. marginatum* tick pools were obtained: 371-Armenia/TI-2022, 372-Armenia/TI-2022, 374-Armenia/TI-2022, 375-Armenia/TI-2022, 229-Armenia/TI-23, 279-Armenia/TI-23, 345-Armenia/TI-23, 423-Armenia/TI-23, and 521-Armenia/TI-23. The sequences were submitted to GenBank under accession numbers PQ878900 - PQ878926.

**Fig 2:**
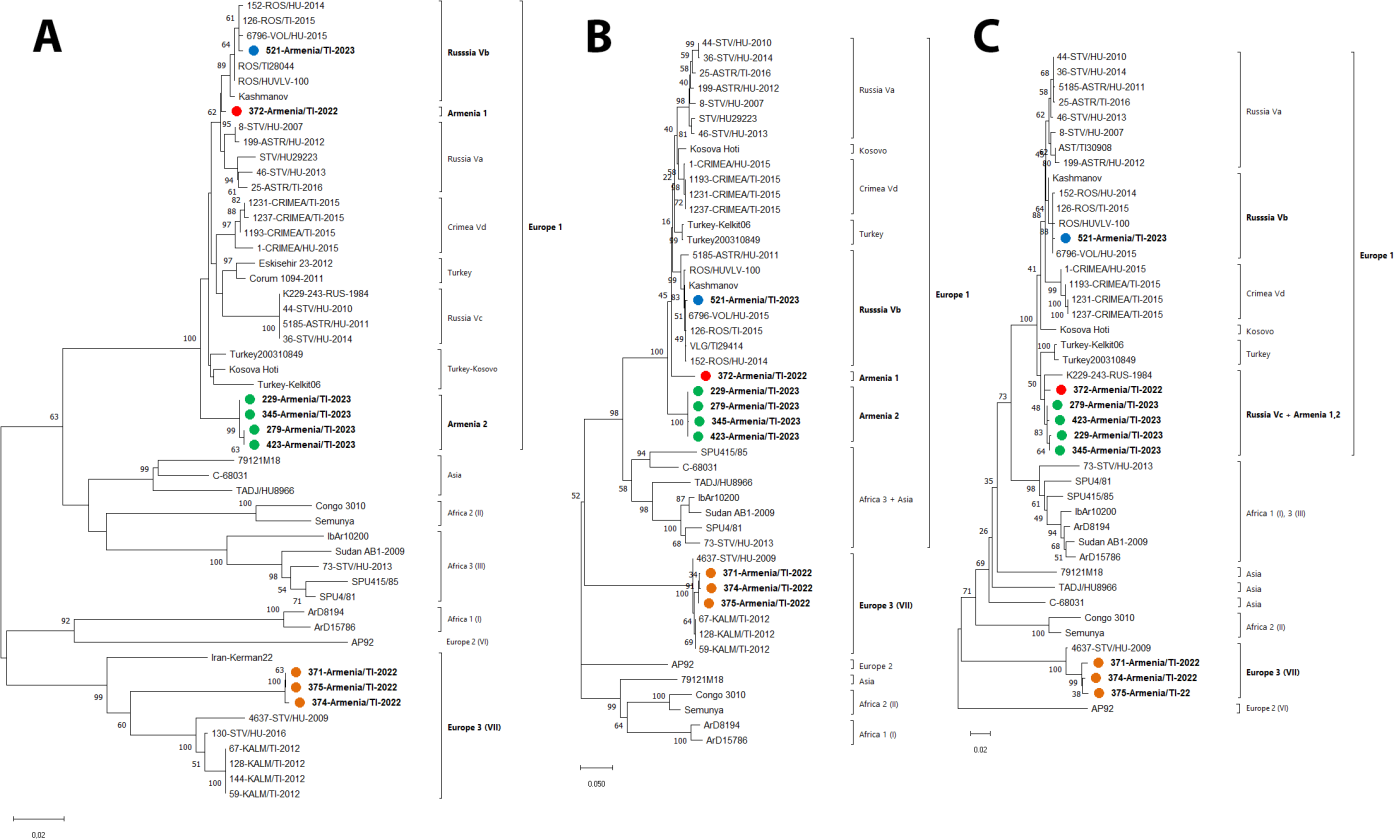
Phylogenetic trees based on viral segments. Key: panel A – S segment (538 bp fragment); panel B – M segment (435 bp fragment); panel C – L segment (437 bp fragment). Sequences from this study are indicated in color.

Phylogenetic trees constructed on the basis of partial S, M, and L segment sequences are presented in Fig 2. The analyzed CCHFV isolates belonged to genetic lineages Europe 1 and Europe 3. On the phylogenetic trees, Armenian CCHFV strains formed two new distinct subgroups within lineage Europe 1 based on S and L segment fragments: Armenia1 (372-Armenia/TI-2022) and Armenia 2 (229-Armenia/TI-23, 345-Armenia/TI-23, 279-Armenia/TI-23, 423-Armenia/TI-23). CCHFV isolate 521-Armenia/TI-23 clustered in the previously described subgroup Volgograd-Rostov-Stavropol (Vb), strains of which are widespread in the natural CCHF focus in the south of Russia.

In the partial L segment tree, the following CCHFV isolates belonged to subgroup Astrakhan-2 (Vc): 372-Armenia/TI-2022, 229-Armenia/TI-23, 345-Armenia/TI-23, 279-Armenia/TI-23, and 423-Armenia/TI-23. Viral isolate 521-Armenia/TI-23 belonged to subgroup Volgograd-Rostov-Stavropol (Vb).

**Fig 3:**
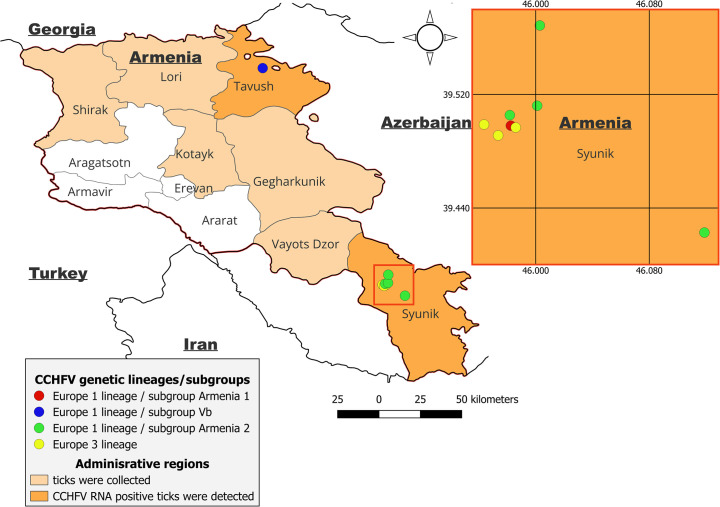
Geographic distribution of CCHFV genetic variants in Armenia. Basemap shapefiles were sourced from GADM (https://geodata.ucdavis.edu/gadm/gadm4.1/shp/).

The geographic distribution of CCHFV genetic variants is shown in Fig 3. Subgroup Vb (Europe 1 lineage) CCHFV circulated in the Tavush region (northern Armenia). Viral strains belonging to the subgroups Armenia 1 and Armenia 2 of the Europe 1 lineage, and also Europe 3 lineage strains, have been detected in the distinct population in the Syunik region (southern Armenia).

### Whole genome sequencing of CCHFV isolates

**Fig 4:**
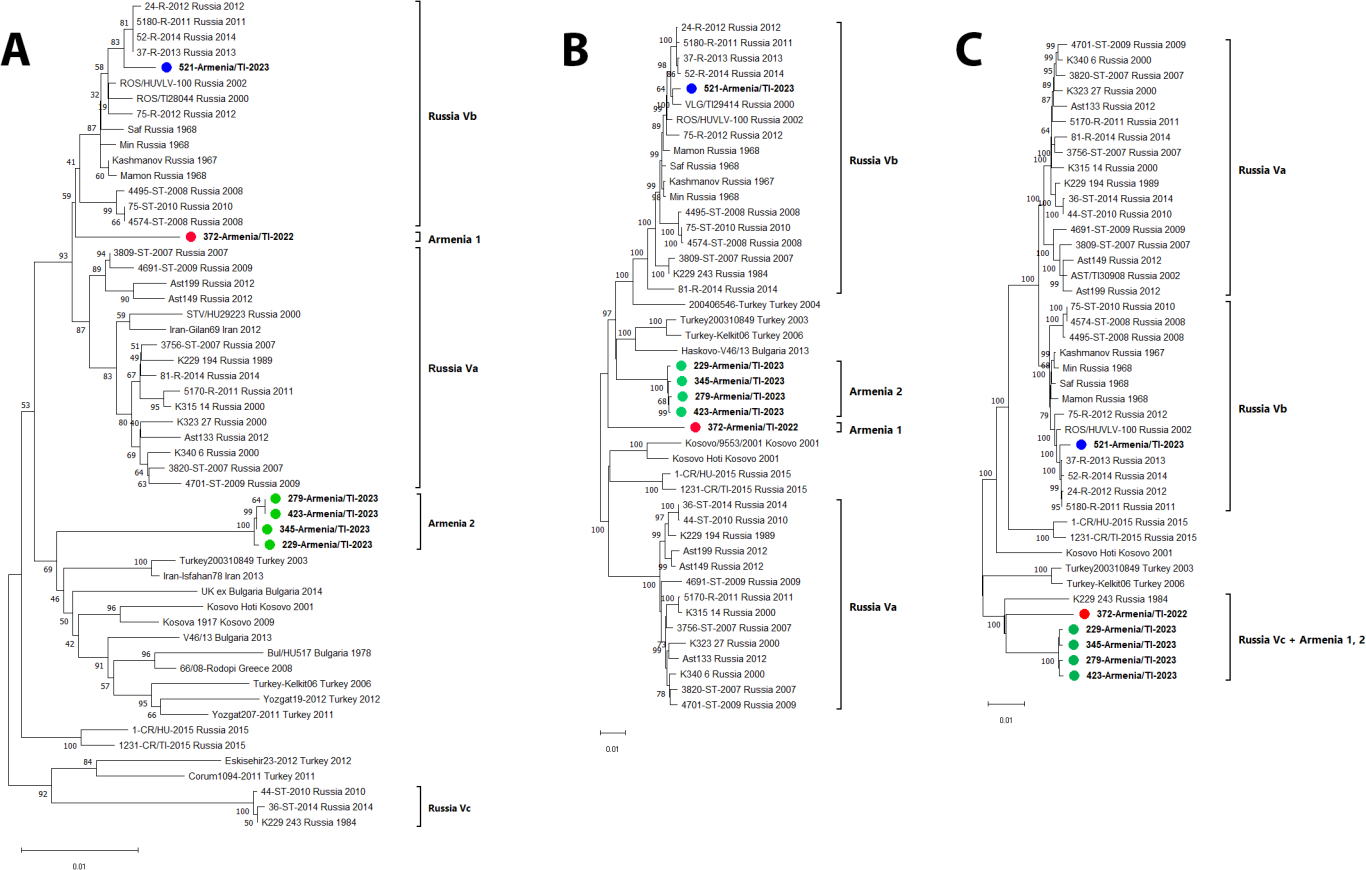
Phylogenetic trees based on viral segment complete ORFs. Key: panel A – S segment; panel B – M segment; panel C – L segment. Sequences from this study are indicated in color.

Whole genome sequencing of six CCHFV isolates was performed: 372-Armenia/TI-22, 229-Armenia/TI-23, 279-Armenia/TI-23, 345-Armenia/TI-23, 423-Armenia/TI-23, and 521-Armenia/TI-23. The obtained sequences were submitted to GenBank (accession numbers PV033786-PV033791, PV091003-PV091008, PV105948-PV105953). The obtained CCHFV isolate sequences (S, M, L segment ORF), and Europe 1 genetic lineage reference sequences, were used for phylogenetic analysis. On the phylogenetic trees based on complete S and L segment ORF, Armenian CCHFV strains belonged to three Europe 1 lineage subgroups: Armenia 1, Armenia 2, Vb (Fig 4). Differences in the topology of phylogenetic trees based on S, M, and L segment coding sequences indicate genomic segment reassortment between CCHFV strains of the Europe 1 genotype.

On the dendrogram by S segment, isolate 372-A/TI-22 of the Armenia 1 subgroup is most genetically similar to Russian viral strains of the Vb subgroup. This isolate is close to Russian and Turkish strains by M segment sequence; it is close to Vc subgroup Russian strains by L segment sequence. The Armenia 2 subgroup isolates are most similar to the strains from Turkey on the dendrograms based on S and M segments; they are most similar to Vc subgroup Russian strains based on L segment.

The Armenia 1 subgroup isolate, relative to other Europe 1 lineage strains, featured: S segment differences (0.29–3.23% nucleotide, 0.45–1.19% amino acid);

M segment differences (4.90–6.44% nucleotide, 3.40–5.44% aa); and

L segment differences (3.28–4.70% nucleotide, 1.11–1.71% aa).

Sequence differences (nucleotide, amino acid) between Armenia 2 subgroup strains and the other Europe 1 lineage strains were noted by: S segment (2.28–4.04%, 0–1.12%); M segment (3.80–5.97%, 3.15–5.07%); and L segment (3.24–4.44%, 1.11–1.74%). Amino acid substitutions specific for strains belonging to the subgroups Armenia 1 (21 substitutions) and Armenia 2 (8 substitutions) were found (Table 2).

**Table 2:** Amino acid substitutions specific for subgroups Armenia 1 and Armenia 2 of the Europe 1 lineage.

Genetic subgroup	S segment	M segment	L segment
Armenia 1	K265G, Y315H	T113A, T119A, E158I, S179L, P192F, L275M, L326I, G335M, I540L	G392R, H762Q, D1885N, Q1982L, T2071I, S2716G, R2879K, T3229A, R3536K, L3864M
Armenia 2	**–**	P122F, P132A, Q1547K, M1586V, F1595S	S1481T, L1683I, S3674C

## Discussion

**Fig 5:**
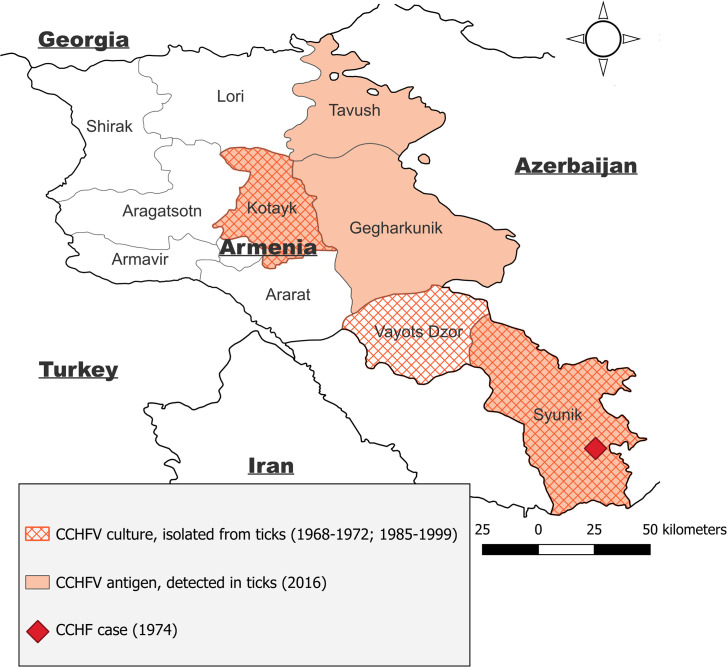
Distribution of CCHFV in Armenia. Retrospective data. Basemap shapefiles were sourced from GADM (https://geodata.ucdavis.edu/gadm/gadm4.1/shp/).

The circulation of CCHFV and other arboviruses was monitored in 1968–1999 in the Republic of Armenia. During that period, CCHFV strains were isolated from pools of ixodid ticks (*H. marginatum, H. anatolicum, R. annulatus, R. bursa, D. marginatus*) collected in the Syunik, Kotayk, and Vayots Dzor regions (Fig 5) [12]. In 1974, a CCHF case was registered in the Syunik region (Sisian district) [14]. In 2016, CCHFV antigen was detected in ticks collected in Armenia [13]. In 2021, CCHFV RNA in *H. marginatum* ticks was found in the country [15].

In 2022–2023, evidence of CCHFV circulation was obtained in the Syunik and Tavush regions. CCHF morbidity was observed previously in these regions alongside diagnostic findings (isolation of CCHFV and viral antigen). The natural focus of CCHF in the RA is probably located in six regions where *H. marginatum* ticks (the main vector for the virus) were found. Several factors support consistent viral circulation in natural CCHF foci in Armenia. One is the large number of *H. marginatum* ticks on farm animals, which exceeds the epidemically significant threshold (abundance index >3.0) [17]. Another is the high prevalence of CCHFV in ticks. These may lead to an increase in the number of CCHF cases in the country. The RA is a recreational zone attractive to tourists. This leads to an increased risk of CCHF case registration in other regions imported from Armenia.

Viral variants of the Europe 1 genetic lineage were detected in the RA, which agrees with the previously established distribution areas of viral genotypes globally. The Europe 1 genotype has previously been identified in the southern part of European Russia, Turkey, Iran, and the countries of southeastern Europe. The CCHFV strains isolated from these counties differ and form distinct genetic subgroups (subtypes) within the Europe 1 lineage [5,6,9–11]. Genetic subgroups Armenia 1 and Armenia 2 within the Europe 1 lineage described in this work include only CCHF viral variants isolated in the RA. Viral strains belonging to subgroup Vb of the Europe 1 lineage were first discovered in Armenia, and this genetic variant is common in European Russia [5].

Viral variants of lineage Europe 3, previously found in Russia and Iran [5,10], were also detected in Armenia. New data on the distribution area of strains belonging to this lineage were obtained.

The obtained data on new CCHF viral variants in Armenia can be used in epidemiological analysis of CCHF cases to differentiate local and imported cases, as well as to improve diagnostic kits for detecting viral RNA. Further studies of CCHFV genetic diversity in Armenia, and other countries of the Transcaucasian region, will allow us: to describe new local genetic variants of virus; and to reconstruct the temporal and spatial distribution of Europe 1 and Europe 3 lineage CCHFV in the region and neighboring countries.

## Supporting information

S1 Table Reference CCHFV nucleotide sequences retrieved from GenBank used in the study. (XLSX)
